# Alcohol use and misuse: What are the contributions of occupation and work organization conditions?

**DOI:** 10.1186/1471-2458-8-333

**Published:** 2008-09-24

**Authors:** Alain Marchand

**Affiliations:** 1 School of Industrial Relations, University of Montreal, Montreal, Canada; 2 Health and Prevention Social Research Group (GRASP), University of Montreal, Montreal, Canada

## Abstract

**Background:**

This research examines the specific contribution of occupation and work organization conditions to alcohol use and misuse. It is based on a social-action model that takes into account agent personality, structures of daily life, and macro social structures.

**Methods:**

Data come from a representative sample of 10,155 workers in Quebec, Canada. Multinomial regression models corrected for sample design effect have been used to predict low-risk and high-risk drinking compared to non-drinkers. The contribution of occupation and work organization conditions (skill used, decision authority, physical and psychological demands, hours worked, irregular work schedule, harassment, unionization, job insecurity, performance pay, prestige) have been adjusted for family situation, social network outside the workplace, and individual characteristics.

**Results:**

Compared to non-qualified blue-collars, both low-risk and high-risk drinking are associated with qualified blue-collars, semi-qualified white-collars, and middle managers; high-risk drinking is associated with upper managers. For constraints-resources related to work organization conditions, only workplace harassment is an important determinant of both low-risk and high-risk drinking, but it is modestly moderated by occupation. Family situation, social support outside work, and personal characteristics of individuals are also associated with alcohol use and misuse. Non-work factors mediated/suppressed the role of occupation and work organization conditions.

**Conclusion:**

Occupation and workplace harassment are important factors associated with alcohol use and misuse. The results support the theoretical model conceptualizing alcohol use and misuse as being the product of stress caused by constraints and resources brought to bear simultaneously by agent personality, structures of daily life, and macro social structures. Occupational alcohol researchers must expand their theoretical perspectives to avoid erroneous conclusions about the specific role of the workplace.

## Background

Alcohol misuse afflicts a substantial part of the working population. In the USA, 6.2% of adults working full-time reported heavy drinking in 1999 [1]. In Canada, 22% to 33% of employees exceeded the Canadian low-risk alcohol guidelines, 7% to 8% were episodic heavy drinkers on a weekly basis [2,3], and 22% reported drinking alcohol at work [4]. Alcohol misuse is of great concern for employers and society since it has been associated with absenteeism and work injuries [3,5-8], as well as with mental health problems like psychological distress [9-16].

Occupation and work organization conditions appear to be mechanisms that explain differentials in worker drinking. However, the contributions of factors outside work, like family situation and personal characteristics, are rarely taken into account in occupational alcohol studies. Consequently, the extent to which occupation and work organization conditions independently contribute to the level of alcohol intake still deserves attention. This paper addresses this issue by examining the contribution of occupation and work organization conditions to patterns of alcohol intake in the workforce. It is based on a social-action model that takes into account agent personality, structures of daily life, and macro social structures.

### Previous studies

According to some studies, variations in alcohol intake are related to position in the occupational structure, as well as to work organization conditions in the workplace. As far as occupation is concerned, managers, blue- and white-collar workers, farmers, and fishermen appear to have more alcohol-related problems than do other workers [6,12,13,17-24]. However, the overall contribution of occupation, per se, seems to be very limited [2,12,13].

As for work organization conditions, occupational and organizational cultural norms, as well as work strain defined relative to alienation and stress [2,12,13,25-29], have been found to be associated with higher levels of alcohol intake. Studies report associations with skill utilization [30,31], decision authority [32-34], physical [35-37] and psychological demands [28,36-39], number of hours worked [17,40], irregular work schedule [4,18], social support at work [34,41], workplace harassment [42], and gratifications [28,43,44].

Beyond the workplace itself, however, some studies have identified contributions from factors linked to family, social network, and individual characteristics. At the family level, being in a couple [18,25,28,41,44-47], having children at home [48], having to cope with work-family conflicts [11,49-51], and household income [51] were associated with variations in alcohol intake. Concerning social networks outside work, having a variety of sources of social support and actively participating in such networks tended to lower alcohol-related problems [33,47]. When it comes to individual characteristics, alcohol intake was more marked among males [12,13,36,41,44-47,52] but decreased with age [28,34,40,45,47,52]. Alcohol intake has also been associated with education [23,25,41], physical health [33], and smoking [28].

The literature reviewed here thus suggests that occupation and work organization conditions could play an important role in alcohol intake and, more broadly, in the problematic use of alcohol. At the same time, though, the literature highlights possible contributions by factors outside the workplace itself and the influence of individual characteristics. However, earlier studies (presented above) have encountered considerable difficulty incorporating, theoretically and empirically, various elements of the social environment (work, family, social networks). Previous studies, moreover, have been unable to include a broad range of workplace conditions to which individuals are subjected in their productive activity, just as they have not been able to take simultaneously into account occupational position and work organization conditions. Most of the studies, moreover, have been gender-specific and male-oriented or conducted on specific occupations, which makes generalizing results to the entire workforce problematic.

### Theoretical model and hypotheses

Alcohol use is not a damaging behaviour per se. Moderate alcohol intake is associated with better cardiovascular health and reduced mortality and may provide psychological benefits such as better subjective health, mood enhancement, stress reduction, sociability, social integration, mental health, long-term cognitive functioning, higher income, and lower absenteeism [36,53]. High levels of alcohol intake lead to reduced benefits, implying that alcohol use and misuse must thus be distinguished analytically.

In order to explain alcohol use and misuse, the theoretical model employed in this study conceptualizes the individual as an agent embedded in a social environment composed of structures with which people have to deal in everyday life. These relationships take place in a social environment defined by social, political, economic, and cultural contexts specific to a given society. The ways people relate to the social environment can be sources of well-being, but also sources of suffering that can affect drinking habits. Social structures and agent personality define conditions of social action [54-57] that place relationships of reciprocity-interaction at the centre of the action, and, together, determine a set of constraints and resources that shapes the contingencies, the locations, and the opportunities to which individuals have access. The relationships between agent and structure can bring about unintended consequences, such that action can lead to results that agents and actors had not sought or anticipated [55,56].

Alcohol use and misuse may be viewed as an unintended consequence of action influenced by the constraints-resources jointly brought to bear by agents and social structures. Constraints may be likened to stressors that have the potential to affect individual capacities for adaptation [58-60] and to cause physiological and mental imbalances [61], whereas resources provide protection to the agent when dealing with stressors in the environment. However, resources do not necessarily prove effective for everyone. In some cases, they may have no effect, whereas in others they may merely allow individuals to reduce the effects of constraints. Constraints-resources imply the existence of both additive and moderating influences on the way stress is experienced [62].

Constraints-resources influencing action come simultaneously from three main levels of social life: macro social structures, structures of daily life, and the personality of agents. Macro social structures are social arrangements tied to the economic, political, and cultural system, as well as to the system of stratification, diversification, and social integration of a society at the national level [57]. It is at this level that the occupational structure arises, which takes the form of a group of positions differentiated by the nature of the work to be accomplished, the tasks carried out, the responsibilities conferred on the individual, and the sector of activity in which the work is performed [63]. Several occupational positions are thus included in a given labour market, among which constraints-resources are unevenly distributed. In this regard, some studies have revealed important variations in work organization conditions by type of occupation [64,65]. This unequal distribution of constraints-resources could give rise to experiences of stress and promote alcohol use and misuse among agents.

Structures of daily life (work, family, social networks) constitute intermediate arrangements between individuals and macro social structures that organize the basis of everyday life, routines, and affective ties [57]. In the workplace, the constraints-resources associated with alcohol use and misuse are linked to four main parameters of work organization [14,15]: task design (skill utilization, decision authority), work demands (physical: from the environment and from efforts made by the individual; psychological: time pressure, quantity, conflict; contractual: hours worked, work schedule), social relations (harassment, unionization), and gratifications (job security, pay, prestige). The effects of constraints-resources at work could also vary according to the position held by the agent in the occupational structure, since this structure influences the distribution of constraints-resources for individuals in the workplace. The effect of working conditions would thus be moderated by the place the agent holds in the occupational structure. As for the links between family and the social network outside the workplace, on the one hand, and alcohol use and misuse, on the other, constraints-resources components define the structure around parental and marital status, strain in marital and parental relationships, household income levels, and the availability of support from the agent's social network to deal with problems issuing from the agent's action in society.

The last level is that of the personality of the agent, which represents the constraints-resources that the individual carries into action, and is related to his/her reflectiveness, rationality, creativity, demographic characteristics, affect, body, biology, representations, perceptions, motivations, habits, and attitudes [54,56,57,66]. The personality of the agent, from a sociological point of view, is not a representation of the individual taken only on the level of traits or personality structure as understood in psychology, but rather an overall representation of conditions characterizing the person that is constructed around the body, the mind, and the social environment [54,67]. To explain alcohol use and misuse, the model thus postulates that characteristics of the personality of the agent are contributing factors and may modify the way work relates to alcohol intake. These characteristics are associated with gender, age, physical health status, lifestyle habits (tobacco use, physical activity), and stressful life events from childhood. This last characteristic might be an interesting factor to analyze given that it has been related to mental health imbalances [68].

Three main hypotheses about the role of the workplace in alcohol use and misuse emerge from this model. H1) Occupational structure and workplace constraints-resources contribute independently to alcohol use and misuse. H2) The effect of workplace constraints-resources on alcohol use and misuse are moderated by the position of the agent in the occupational structure. H3) Agent personality, family, and social network outside work modulate the effects of workplace constraints-resources on alcohol use and misuse.

Overall, these hypotheses assume associations to be equivalent for both genders. However, if we assume differentials in drinking and an unequal distribution of occupations and work-organization conditions between genders, we must then evaluate any moderating effect that gender might have on the relationship between work and alcohol use and misuse.

## Methods

### Data

The data are derived from the Québec Health and Social Survey (QHSS) conducted in 1998 [69] by the Institut de la statistique du Québec (Government of Québec statistical Institute). This cross-sectional survey provided a representative sample of the Québec population and was based on a complex sampling design of 11,986 randomly selected households (response rate = 82.1%). For each household, all the members aged 15 and over were invited to fill out a questionnaire relating to health and socioeconomic indicators. Informed written consent was obtained for each participant. The survey was completed by 20,773 respondents (response rate = 84.0%), for an overall response rate of 69% (82.1%*84.0%). The data were weighted to adjust for selection probability and response rate, as well as for demographic distribution by gender, age, and region according to the 1996 Canadian census. After deletion of cases with missing values, the QHSS-98 comprised useable responses from 10,155 employed persons. Women made up 44.3% of the respondents. Average respondent age fell into the 35–39 age group.

### Measures

#### Alcohol use and misuse

Type of drinker categories were used to measure alcohol use and misuse. Respondents indicated the number of drinks they had had on each day during the week preceding questionnaire administration. Respondents were then classified into three categories: 1) abstainers (at least during the previous seven days); 2) low-risk drinkers: no more than 9 drinks for females and no more than 14 drinks for males in the previous seven days; and 3) high-risk drinkers: 10 drinks or more for females, 15 drinks or more for males in the previous seven days. The cut-off points used for differentiating low- and high-risk drinkers on the basis of weekly consumption volume follow the Canadian guidelines for low-risk consumption [70]. For non-drinkers, we initially distinguished those who were abstainers over their lifetimes or for at least one year from those who were drinkers but abstained during the week preceding the survey. Lifetime abstainers and former drinkers with at least one year of abstention were classified in the same category (abstainers) because the proportion of respondents in each category was small, because former drinkers had periods of abstention that averaged 9.29 years, and because there was no difference between lifetime abstainers and former drinkers in the prevalence of mental health problems [13]. Furthermore, people who were drinkers but abstained the week preceding the survey were also merged with abstainers because, compared to low-risk and high-risk drinkers, no differences were found in the variables for work, family, and social support outside the workplace.

#### Occupations

Occupations were recorded according to the 4-digit codes of the Canadian Classification and Dictionary of Occupations [71]. This classification described individual occupations according to actual tasks performed at work, the responsibilities attached to the job, and the type of industry or sector within which the work was carried out. Overall, 482 occupations were reported by the QHSS, which were recoded according to the sixteen larger professional categories of the Canadian Socio-economic Classification of Occupations [72]. These categories were assumed to be sociologically homogeneous in prestige, income, and schooling. The classification distinguished three levels of qualifications for blue-collar and white-collar occupations. To adjust for the small sample sizes in some categories, self-employed professionals and employed professionals were grouped in a single category, as was done for foremen and supervisors. Farmers were clustered with qualified blue-collar workers, as were farm labourers with non-qualified blue-collars.

#### Task design

Skill utilization and decision authority were derived from the complete and validated French-language version [73,74] of the Karasek [75] Job Content Questionnaire (JCQ). Based on a 4-point Likert scale (disagree/agree), skill utilization was obtained by summing six items (alpha = 0.81; e.g., my job requires that I learn new things) and decision authority by summing three items (alpha = 0.81; e.g., my job allows me to make a lot of decisions on my own).

#### Work demands

The scale for physical demands was constructed from the summation of 10 items associated with the environment and with the level of individual physical effort [13]. Each item was measured on a 4-point Likert scale (never/all the time) (alpha = 0.82). Psychological demands (alpha = 0.69) were based on the sum of nine 4-point indicators (disagree/agree) of the complete French version of the Karasek [75] JQC (e.g., my job requires working very fast). For contractual demands, working hours were measured by adding the number of hours spent on the main job and, if applicable, on other jobs. Work schedule irregularity was based on a 4-point Likert scale (never/all the time) measuring the frequency of respondent exposure to irregular or unpredictable schedules.

#### Social relations at work

Workplace harassment was used as an indicator of the nature of the problems faced by workers when interacting with colleagues and superiors; unionization was considered a proxy for resources that could be available in the workplace to support workers when problems were encountered. Workplace harassment was measured using three 4-point Likert indicators (never/very often). Respondents were asked to indicate whether, during the previous 12 months, they had been subjected to physical violence or intimidation and/or had been the objects of unwelcome remarks or actions of a sexual nature in the workplace. The variable was coded 1 for respondents reporting at least one situation (occasionally, often, or very often), and coded 0 for the others. Unionization status contrasted respondents reporting union membership (coded 1) with those not belonging to unions (coded 0).

#### Gratifications

Job insecurity was measured by a dichotomous variable where 0 referred to a permanent job and 1 a temporary job (temporary job with a set termination date, temporary job without a set termination date, and other type of job). Performance pay designated frequency for pay based on performance, commissions, or piece work using a 4-point Likert scale (never/all the time). Prestige was determined using the Blishen index [76].

#### Family situation and social network

Marital status contrasted respondents living together as a couple (coded 1) with other marital situations (coded 0). Parental status was measured by the number of minor children (under 18 years of age) living with the respondent. Household income was determined using a 5-point ordinal scale (very poor/upper income) from the Québec Statistics Institute, which measured the level of sufficiency of income in relation to household size. Marital stress was based on an additive scale (alpha = 0.72) of three items (true/false) developed by Wheaton [60]: your partner doesn't understand you; your partner doesn't show enough affection; and your partner is not committed enough to your relationship. Strained parent-child relations were measured by a 5-point scale (no problem/constant problems) on which values corresponded to the frequency of problems experienced by the respondent with natural-born minor children and/or non-natural-born children. Social support outside the workplace included three items measuring the number of people on whom the respondent could rely: the presence of a person with whom one could speak openly about problems; the presence of a person in one's circle of family or friends who could offer help if needed; and the presence of a person in one's circle of family or friends with whom the respondent felt a closeness and who displayed affection (alpha = 0.73).

#### Demography and physical health

Gender was a dichotomous variable coded 0 for male and 1 for female. Age was measured on an ordinal scale that included 12 categories ranging from 15 years old to 65 years old and over. Physical health status was based on the number of physical health problems experienced by the respondent at the time of the survey, using a list of 27 health problems such as heart disease, cancer, arthritis, emphysema, etc. The scale had a range of 0 to 3, where 3 indicated 3 problems or more.

#### Lifestyle habits and stressful childhood events

Alcohol consumption was based on the total number of glasses consumed daily during the previous 7 days; those who had not consumed during the previous week were coded as 0. This measure was used in its logarithmic (ln) form to correct for the asymmetry of the original distribution. Cigarette use was measured by the number of cigarettes smoked per day. Physical activity was determined by the frequency of participation in a physical activity lasting more than 20 to 30 minutes during the three preceding months using a 7-point scale (none/4 times or more a week). Stressful childhood events included 7 yes/no items developed by Wheaton [60] for events that occurred before age 18 (e.g., spending two weeks or more in hospital; parents divorcing; alpha = 0.54).

Table 1 presents descriptive statistics for the overall sample.

**Table 1:** QHSS-1998 descriptive statistics (n = 10,155)

	Min-Max	Mean/percentage	Standard deviation
**ALCOHOL USE AND MISUSE** Non-drinker	0–1	38%	-
Low-risk drinker	0–1	51%	-
High-risk drinker	0–1	11%	-
**OCCUPATIONS** Upper managers	0–1	3%	-
Middle managers	0–1	8%	-
Front-line supervisors	0–1	6%	-
Professionals	0–1	11%	-
Semi-professionals	0–1	9%	-
Technicians	0–1	2%	-
Qualified white-collars	0–1	9%	-
Semi-qualified white-collars	0–1	16%	-
Non-qualified white-collars	0–1	5%	-
Qualified blue-collars	0–1	9%	-
Semi-qualified blue-collars	0–1	10%	-
Non-qualified blue-collars	0–1	11%	-
**TASK DESIGN** Skill utilization	6–24	17.59	3.63
Decision authority	3–12	8.73	2.16
**DEMANDS** Physical demands	10–40	13.10	4.31
Psychological demands	9–36	22.70	4.20
Hours worked	15–110	38.39	12.15
Irregular work schedule	1–4	2.07	1.03
**SOCIAL RELATIONS** Harassment	0–1	20%	-
Unionization	0–1	34%	-
**GRATIFICATIONS** Job insecurity	0–1	0.15	0.35
Performance pay	1–4	1.30	0.82
Prestige	17.7–101.7	43.46	14.33
**FAMILY** Marital status (living together)	0–1	69%	-
Number of minor children	0–3	0.63	0.92
Household income	1–5	3.69	0.89
Strained marital relations	0–3	0.33	0.73
Strained parental relations	1–5	1.29	0.65
**SOCIAL NETWORK** Social support (outside work)	0–015	8.80	3.82
**AGENT** Gender (female)	0–1	44%	-
Age	15–17/65+	35–39	8.59–9.60
Physical health (number)	0–3	0.77	0.96
Cigarettes (per week)	0–85	5.93	10.44
Physical activities (frequency)	1–7	3.58	2.09
Stressful childhood events (number)	0–7	0.88	1.16

### Analysis

Because alcohol use and misuse is a nominal variable with three categories, multinomial regression models were estimated using abstainers as the reference category. Since the sample analyzed here is based on a complex sampling design, standard errors for hypothesis testing and 95% confidence-interval computations were corrected for the design effect (robust standard errors) generated by the clustering of observations at the household level. In QHSS-98, the global design effect (deft) is estimated at 1.41, meaning that standard errors and Chi-square tests need to be deflated by an amount of 41%. While the sample size is large (n = 10 155), the effective sample size net of design effect is n = 5992. Parameter estimations were obtained using maximum likelihood estimation with robust standard errors available in Mplus 4.21 [77]. Satorra-Bentler scaled Chi-square tests corrected for design effects were used for evaluating the significance of each variable, interaction terms, and the overall fit of the models [77,78]. The Satorra-Bentler scaled chi-square used a scaling correction to better approximate chi-square under non-normality. The probability of rejection of the null hypothesis was set at p = 0.05. Continuous independent variables were tested to determine the existence of non-linear relationships (quadratic term tested), and none of them were found to deviate significantly from linearity.

Several multinomial regressions models were estimated. In the first model, the variables concerning occupation and the work organization conditions were introduced into the equation in order to verify their unadjusted contributions to alcohol use and misuse. In the second model, the variables describing family, social network, and the personality of the agent were entered together, in order to determine whether the effects of occupation and the workplace were modulated by the other structures of daily life and/or the personality of the agent. This sequencing of models allowed us to test the first (H1) and the third (H3) hypothesis. Third, the interactions for each work organization condition and occupations were evaluated in order to establish their role in the explanation of alcohol use and misuse (H2). Last, the interactions for occupations by gender, and work organization conditions by gender were estimated separately. For steps three and four, interaction terms were constructed using multiplication of elements and chi-square tested for significance.

## Results

Table 2 and 3 present the results of two multinomial regression models. Table 2 reports chi-square tests for the significance of each variable in the models, and Table 3 presents odds-ratios and 95% intervals. In Model 1, the unadjusted effects of occupation and workplace variables indicate statistical significance. Compared to non-qualified blue-collars, being a qualified blue-collar and being a victim of workplace harassment are associated with both low- and high-risk drinking. Skill utilization and prestige are correlated with low-risk drinkers and decision authority with high-risk drinkers.

**Table 2:** Chi-square tests for each variable in the multinomial regression models of alcohol use and misuse

	**Model 1**		**Model 2**	
	**χ**^2^	**df**	**χ**^2^	**df**
**OCCUPATIONS**	41.31**	22	44.49**	22
**TASK DESIGN** Skill utilization	5.98*	2	4.75	2
Decision authority	6.56*	2	3.06	2
**DEMANDS** Physical demands	2.21	2	1.61	2
Psychological demands	0.92	2	1.55	2
Hours worked	1.50	2	0.89	2
Irregular work schedule	1.32	2	1.74	2
**SOCIAL RELATIONS** Harassment	25.26**	2	26.08**	2
Unionization	1.79	2	4.44	2
**GRATIFICATIONS** Job insecurity	1.20	2	0.56	2
Performance pay	3.88	2	2.63	2
Prestige	11.78**	2	2.70	2
**FAMILY** Marital status (living together)			15.40**	2
Number of minor children			22.46**	2
Household income			53.00**	2
Strained marital relations			2.61	2
Strained parental relations			11.70**	2
**SOCIAL NETWORK** Social support (outside work)			21.49**	2
**AGENT** Gender (female)			75.76**	2
Age			4.06	2
Physical health			4.27	2
Cigarettes			66.50**	2
Physical activities			6.94*	2
Stressful childhood events			1.50	2

Note: * p ≤ .0.05; ** p ≤ .01

**Table 3:** Odds-ratios and 95% confidence intervals for the multinomial regression models of alcohol use and misuse

	**Model 1**				**Model 2**			
	**Low-risk^1^** **n = 5179**		**High-risk^1^** **n = 1117**		**Low-risk^1^** **n = 5179**		**High-risk^1^** **n = 1117**	
	**OR**	**95% CI**	**OR**	**95% CI**	**OR**	**95% CI**	**OR**	**95% CI**
**OCCUPATIONS^2^** Upper managers	0.96	0.52–1.77	1.41	0.64–3.11	1.35	0.75–2.45	2.39*	1.07–5.34
Middle managers	1.47	0.99–2.20	1.33	0.77–2.32	1.84**	1.24–2.75	1.87*	1.05–3.32
Front-line supervisors	1.12	0.81–1.55	1.05	0.65–1.70	1.28	0.92–1.77	1.44	0.88–2.37
Professionals	0.94	0.60–1.49	0.86	0.45–1.65	1.38	0.87–2.19	1.67	0.85–3.31
Semi-professionals	1.03	0.69–1.52	1.07	0.61–1.89	1.32	0.89–1.96	1.70	0.94–3.07
Technicians	1.26	0.78–2.04	1.07	0.53–2.17	1.53	0.94–2.48	1.60	0.75–3.41
Qualified white-collars	0.93	0.69–1.26	0.73	0.45–1.18	1.26	0.92–1.72	1.18	0.71–1.97
Semi-qualified white-collars	1.19	0.95–1.50	1.30	0.91–1.86	1.44**	1.13–1.83	1.71**	1.18–2.49
Non-qualified white-collars	1.07	0.80–1.42	0.87	0.52–1.48	1.34	0.99–1.81	1.27	0.74–2.19
Qualified blue-collars	1.38*	1.05–1.81	1.73**	1.16–2.58	1.48**	1.13–1.95	2.11*	1.41–3.16
Semi-qualified blue-collars	1.00	0.79–1.25	1.05	0.70–1.56	1.08	0.86–1.36	1.21	0.81–1.81
**TASK DESIGN** Skill utilization	1.02*	1.00–1.05	1.03	0.99–1.07	1.02	1.00–1.04	1.03	0.99–1.06
Decision authority	1.03	0.99–1.06	1.07**	1.01–1.12	1.02	0.98–1.05	1.05	0.99–1.10
**DEMANDS** Physical demands	1.00	0.99–1.02	1.02	0.99–1.04	0.99	0.97–1.01	1.00	0.98–1.03
Psychological demands	1.00	0.98–1.01	0.99	0.96–1.01	1.00	0.98–1.02	0.98	0.96–1.01
Hours worked	1.00	1.00–1.01	1.00	1.00–1.01	1.00	0.99–1.00	1.00	0.99–1.00
Irregular work schedule	1.04	0.98–1.10	1.00	0.90–1.10	1.03	0.97–1.10	0.97	0.88–1.07
**SOCIAL RELATIONS** Harassment	1.18*	1.02–1.37	1.76**	1.42–2.20	1.27**	1.09–1.47	1.78**	1.42–2.24
Unionization	0.96	0.84–1.09	0.89	0.72–1.09	0.90	0.79–1.03	0.83	0.68–1.03
**GRATIFICATIONS** Job insecurity	0.92	0.78–1.09	0.98	0.76–1.27	0.95	0.80–1.12	0.97	0.74–1.27
Performance pay	1.03	0.96–1.10	1.10	1.00–1.22	1.01	0.94–1.08	1.07	0.96–1.19
Prestige	1.02**	1.01–1.03	1.01	1.00–1.03	1.01	0.99–1.02	1.00	0.99–1.02
**FAMILY** Marital status (living together)					0.83*	0.71–0.97	0.64**	0.50–0.81
Number of minor children					1.10*	1.02–1.20	0.80**	0.68–0.93
Household income					1.26**	1.17–1.36	1.41**	1.24–1.61
Strained marital relations					0.97	0.90–1.05	1.09	0.95–1.25
Strained parental relations					1.05	0.95–1.17	1.33**	1.13–1.57
**SOCIAL NETWORK** Social support (outside work)					1.02**	1.01–1.04	1.06**	1.03–1.09
**AGENT** Gender (female)					0.57**	0.49–0.65	0.48**	0.39–0.60
Age					1.03	1.00–1.06	1.02	0.97–1.07
Physical health					0.94	0.89–1.00	0.95	0.86–1.04
Cigarettes					1.00	0.99–1.00	1.03**	1.02–1.04
Physical activities					1.04*	1.01–1.07	1.04	0.99–1.08
Stressful childhood events					0.99	0.94–1.04	1.02	0.95–1.11

χ^2 ^(df)	220.28 (44)**				511.11 (68)**			

Note: * p ≤ .0.05; ** p ≤ .01. 1) Reference is non-drinkers. 2) Reference is non-qualified blue-collars

Model 2 looks at the personality of the agent and other structures of daily life (family, social network) as mediators of work factors. Overall, this model gives support to the first hypothesis (H1). It shows that qualified blue-collar workers and workplace harassment still contribute to type of drinker. However, the associations among skill utilization, decision authority, and prestige are mediated by agent personality, family, and social network outside the workplace, thus supporting the third hypothesis (H3). Furthermore, and lending added support to the third hypothesis (H3), the introduction of these latter factors into the model adds suppressive effects because new occupations appear to be related to the outcome. Upper managers are now associated with high-risk drinking, and front-line supervisors and semi-qualified white-collars are also now correlated with both low-risk and high-risk drinking. Finally, gender, marital status, number of minor children, household income, and social support outside the workplace contribute to both low-risk and high-risk drinking. Smoking is related only to high-risk drinking and physical activities only to low-risk drinking.

Based on Model 2, tests of the interaction between occupations and workplace variables revealed that only the interaction between occupation and harassment, which gives modest support to the second hypothesis (H2), is significant (χ2 = 36.71, df = 22, p = 0.025). In Model 2, the overall effect of harassment on high-risk drinking is OR = 1.78, and the interactions reveal stronger associations for non-qualified blue-collars (OR = 2.95) and semi-professionals (OR = 2.21). The association is smaller for non-qualified white-collars (OR = 1.28) and non-significant for technicians (OR = 0.97).

In the final analysis, the interactions between work and gender are non-significant. The interaction between gender and occupation reaches χ2 = 14.44, df = 22, p = 0.885 and χ2 = 5.65, df = 20, p = 0.999 for gender by work organization conditions.

## Discussion

This study has examined the specific contributions of occupation and work organization conditions to alcohol use and misuse among workers. The findings support the hypothesis that position in the occupational structure, and to a lesser extent workplace constraints-resources, are associated with individual alcohol intake beyond the contributions of family situation, social support outside the workplace, and personal characteristics. The effect of workplace constraints-resources did not vary with position in the occupational structure, with the sole exception of workplace harassment. Nor were the effects of occupation and work organization conditions a function of gender. The results confirm the relevance of a theoretical model that elaborates on the problem of alcohol use and misuse as being the product of stress caused by the constraints and the resources brought to bear simultaneously by agent personality, structures of daily life, and macro social structures.

At the macro social structures level, occupation appears to be an important predictor of alcohol use and misuse, and has a greater influence than work organization conditions themselves. Low-risk drinking is not, per se, a damaging condition. Middle managers, semi-qualified white-collars, and qualified blue-collars had 44% to 85% greater odds of being low-risk drinkers than did non-qualified blue-collars. For some of these workers, alcohol could be used as a tension-reduction mechanism associated with pleasure and well-being [36,53]. However, for other workers in the same occupations, the stress experienced may be so high that increases in alcohol consumption act as a tension-reduction mechanism associated more with reduced benefits. For these workers, the odds of high-risk drinking increased from 71% to 111%. It is noteworthy that upper mangers also were a high-risk group, given that rates of high-risk drinking were 139% higher among upper managers.

Overall, these results parallel those obtained in previous research [6,12,13,17-24], but the contribution of occupation seems stronger compared to what was previously reported [2,12]. Furthermore, occupations could also play an indirect role in stimulating alcohol use and misuse, given the unequal distribution of work organization conditions by type of occupation [64,65]. This uneven distribution raises questions about how occupational structure could also act indirectly on alcohol use and misuse by conditioning constraints-resources in the family, social network, and agent personality. Based on the results obtained here, occupation may moderate the relationship between social relations at work and alcohol intake because occupation may interact with workplace harassment and high-risk drinking. However, the moderating role of occupation is very limited and thus only partially supports the second hypothesis (H2).

The stress levels generated by the constraints-resources of the structures of daily life have also clearly proved important for understanding variations in individual alcohol use and misuse. However, the contribution of work organization conditions in explaining this outcome appears very small when the analysis takes into account occupation, other structures of daily life, and individual characteristics. Using such a model, only workplace harassment, as has also been found elsewhere [42], shows itself to be associated with 27% greater odds of low-risk drinking and 78% greater odds of high-risk drinking. However, although the association with high-risk drinking is itself moderated by occupation, this result has only borderline significance. The results obtained here thus do not support the contentions of earlier studies reporting associations between many aspects of work organization conditions and alcohol intake. This absence of corroboration exists because previous studies did not take simultaneous account of occupation, family, social network, and diversity of individual characteristics [4,17,18,28,30-41,43].

The data support the theoretical model concerning the potentially stressful role of constraints-resources for the other structures of daily life, which include family and social network outside the workplace. At the family level, being in a couple reduces the odds of both low-risk and high-risk drinking; the association is stronger for high-risk drinking. However, the number of minor children in the household affects this association according to type drinker. For each additional child, the odds of low-risk drinking increases by 8%, but the odds of high-risk drinking decreases by 20%. By contrast, if strained parental relations prevail among workers, the odds of high-risk drinking goes up by 33%. For its part, household income sufficiency is related to both types of drinking; the effect is stronger for high-risk drinkers. As for the social network outside the workplace, the results show an increment in the odds of both low-risk and high-risk drinking, which seems to contradict what has been reported in earlier studies [33,47] that employed limited conceptual frameworks.

Last, the results confirm the role of the personality of the agent in the theoretical model. This variable acts to complement structures of daily life and macro social structures for understanding alcohol use and misuse in the workforce. The results show that low-risk and high-risk drinking are greater among men; smoking increases the odds of high-risk drinking; and physical activities are associated with low-risk drinking. The analysis carried out here, however, does not support previous research reporting effects for age [28,34,40,45,47,52] and physical health [33] when occupation, work organization conditions, family situation, and the social network outside the workplace place are taken into account. Likewise, when used as an exploratory variable, stressful childhood events are not significant.

Overall, the results are clearly supportive of the third hypothesis (H3). Agent personality, family, and social network outside the workplace mediate and suppress [79,80] the effects of certain occupations and workplace-organization conditions. Mediation can be observed for skill utilization, decision authority, and occupational prestige. These results suggest that task design and certain gratifications at work do not seem to contribute directly to alcohol intake when a broader conceptual framework capturing the complexity of individual action is used. However, this broader framework also captures the effects of other occupations that do not show up in the unadjusted model because suppression effects are revealed for upper managers (high-risk drinking), and for front-line supervisors and semi-qualified white-collars (low-risk and high-risk drinking).

Finally, the results do not support moderating effects associated with gender. When the model includes the diversity of worker constraints-resources that occur in macro social structures, structures of daily life, and agent personality, the associations between occupation and alcohol drinking and between work organization conditions and drinking are the same for males and females. This finding is consistent with previous studies reporting non-significant gender interactions [2,12] and more broadly with recent studies showing no gender-associated differentials for occupation, work stressors, and mental health [14,15,81-83].

The present study nevertheless has limitations. First, the data are cross-sectional, which implies that the relationships observed cannot be interpreted causally and will need to be replicated longitudinally. Second, the fact that the analyses are limited to available QHSS indicators implies that variables such as social support in the workplace, and alcohol norms related to occupational and organizational culture, were not measured, whereas they have been linked to alcohol intake in other studies. Third, the QHSS did not take into account workplace factors having to do with management and supervisory styles or occupational health and safety resources. These elements are potentially important determinants of quality of life and workplace well-being that can be associated with alcohol use and misuse. Fourth, individual alcohol consumption data reported across seven days in the QHSS-1987 did not specify the pattern of consumption over the seven days. It is therefore impossible to distinguish consumption during weekends from that during the rest of the week. It is reasonable to expect that, in general, alcohol intake is greater during weekends, whereas among individuals experiencing psychological distress, consumption patterns might be consistently higher for all days of the week.

Fifth, measurements are assumed to be free of estimation errors, which is almost never the case with auto-reported data. This is particularly true for alcohol intake, which has been found to be under-reported [84,85]. Sixth, the hypothesis that an occupation-selection bias exists, such that workers might choose occupations on the basis of their expectations about the acceptability of certain alcohol-intake levels, could not be rejected. Only a longitudinal study could address this question. However, one could easily argue that the main process motivating individual choice of occupation is more likely to be based primarily on the adequacy of personal resources for meeting job requirements (e.g., schooling, experience, qualifications, personality-based expectations). Last, while the large sample size increased the power of the study and by the same token increased the odds of finding significant variables having very small association with the outcome, correction for design effects by an amount of 41% guarded against this potential problem.

## Conclusion

Based on the available data in the QHSS, this study suggests that occupation and social relations at work that yield to harassment are, in and of themselves, important factors associated with alcohol use and misuse in the workforce. Confirming the theoretical model, which posits a combined role for stress emerging from constraints-resources embedded in macro social structures, structures of daily life, and agent personality, will require expanding future research approaches to the study of alcohol in the workforce to avoid erroneous conclusions about the role of the workplace. Integrating family situation, support from the social network outside the workplace, and a diversity of individual characteristics will help identify dynamics inherent in occupation and workplaces that manifest as alcohol-related problems. However, further research will be needed to specify how firms create constraints-resources for agents. Constraints-resources vary from company to company, and indeed from department to department within companies. Management structures and styles, as well as the mechanisms set up by organizations to coordinate production with human resources priorities, unquestionably constitute factors worthy of more study because they can predispose workers to alcohol use and particularly alcohol misuse in the work environment.

## Competing interests

The author declares that they have no competing interests.

## Authors' contributions

AM contributed to data analysis and the writing of the paper. AM read and approved the final manuscript.

## Pre-publication history

The pre-publication history for this paper can be accessed here:


